# Age-related differences in linear sprint in adolescent female soccer players

**DOI:** 10.1186/s13102-021-00327-8

**Published:** 2021-08-22

**Authors:** Elena Mainer-Pardos, Oliver Gonzalo-Skok, Hadi Nobari, Demetrio Lozano, Jorge Pérez-Gómez

**Affiliations:** 1 Health Sciences Faculty, Universidad San Jorge, Autov A23 km 299, 50830 Villanueva de Gállego, Zaragoza Spain; 2 Head of Return To Play, 41005 Sevilla, FC Spain; 3 Department of Exercise Physiology, Faculty of Sport Sciences, University of Isfahan, 81746-7344 Isfahan, Iran; 4 Sports Scientist, Sepahan Football Club, 81887-78473 Isfahan, Iran; 5 HEME Research Group, Faculty of Sport Sciences, University of Extremadura, 10003 Cáceres, Spain

**Keywords:** Football, Velocity, Anthropometric, Performance, Maturation, Youth sports

## Abstract

**Background:**

Several studies have observed the contribution of chronological age, biological maturation, and anthropometric characteristics to sprinting performance in young soccer players. Nevertheless, there are no studies that have analysed the contribution of these characteristics to running speed qualities in adolescent female soccer players.

**Objective:**

This study investigated age-related differences in sprint performance in adolescent female soccer players. Also, it examined the possible influence of anthropometry [body mass and body mass index (BMI)] and biological maturation [age at peak height velocity (APHV)] in sprint performance.

**Methods:**

Eighty adolescent female soccer players [under (U) 14, n = 20; U16, n = 37; U18, n = 23] participated in this study. Players were tested for 40 m sprint (each 10 m split times).

**Results:**

*Posthoc* analysis revealed better performance in all split sprint times of older soccer players (U18 and U16) compared with younger category (F: 3.380 to 6.169; *p* < 0.05; ES: 0.64 to 1.33). On the contrary in all split sprint times, there were no significant changes between U16 and U18 (*p* < 0.05; ES: 0.03 to 0.17). ANCOVA revealed differences in all parameters between groups, controlled for APHV (*p* < 0.05). In contrast, all between-group differences disappeared after body mass and BMI adjustment (*p* > 0.05). Finally, the results indicate that BMI and body mass were significantly correlated with 40 m sprint (*p* < 0.05; *r*: -0.31) and 20 m flying (*p* < 0.01; r: 0.38), respectively.

**Conclusion:**

In the present players’ sample, body mass and BMI had a significant impact on running speed qualities.

## Introduction

Women’s soccer has increased in popularity and participation during the last decade [1]. Fédération Internationale de Football Association (FIFA), in the last report, estimates that there is close to one million of under-18 (U-18) players in Europe, having increased the number of licenses in just one year by more than 10% [1]. So, nowadays, there is an increase of studies and research in this population due to the growth of this sport at early ages.

In soccer, speed is considered a main determinant of performance [2, 3] and it is the most dominant action when scoring goals in young players from 13 to 16 years [4]. Therefore, the measure of straight sprinting could in part detect if someone continue a career as a professional or amateur player in adulthood. Despite increasing the popularity of soccer among female adolescents, there is a little information about the possible effect of age on sprint performance in this population.

Soccer is considered a contact sport and demands a wide variety of skills at different intensities [5, 6]. The ability to perform high-speed actions is an important prerequisite to successfully perform different actions in soccer [2, 7]. Several studies with soccer players have shown that the improvement of high-speed components such as acceleration [8, 9] and maximum running speed [10–12] are related to match performance. Usually, this quality is associated with a successful performance on soccer match play [13]. The ability to accelerate is a skill that manifests itself in short distances [2], specifically, it is divided into the initial acceleration in the first 5 to 10 m [14] and maximum running speed likely reached after 20 to 40 m [15, 16]. However, concerning the distance of sprint, Valquer et al. [17] observed that 96% of sprints during a match are shorter than 30 m, with 49% of them being shorter than 10 m. So the ability to accelerate and maximum running speed are crucial in game performance. For this reason, coaches should take into account the development of these abilities relative to the age [18] and their stage of maturation [19–21].

Biological maturation is a gradual process over time in which modifications are presented at the anatomical and physiological level. Hormones are stimulated during the period of puberty until full maturity is reached [22]. The increase in circulating hormonal levels improves neural function, coordination andpower and also produces both oxidative and non-oxidative changes in the metabolism [21, 22]. Any inappropriate stimulus could cause some alteration in the development of the adolescent and that is why, the training loads should adapt to the speed of subject’s maturation [23]. Several studies have observed the contribution of chronological age, biological maturation and anthropometric characteristics to sprinting performance in young soccer players [16, 20, 24]. For example, Malina et al. [24] showed that height, body mass and maturity status are determinants of sprint-running performance. Emmonds et al. demonstrated that anthropometric and performance characteristics develop with increasing age in high-level youth female soccer players [25]. These differences in anthropometric parameters are attributed to increased maturity, biological, hormonal and neurological changes [25]. As such, this scarce information makes it challenging to provide solid, evidence based recommendations on the contribution of these characteristics to sprint performance in adolescent female soccer players.

Thus, the aims of this study were: 1) to investigate age related differences in sprint performance in different age groups of adolescent female soccer players (U14 to U18) and, 2) to examine the influence of body mass, body mass index (BMI) and biological maturation on sprint performance.

## Material and method

### Selection of study groups

Eighty female soccer players (U14, U16 and U18) belonging to the same female soccer division club academy squad (Iberdrola Women's First Division) participated in this study (age: 14.9 ± 1.5 year; height: 158.6 ± 6.7 cm; body mass: 53.3 ± 7.8 kg; body mass index (BMI): 21.1 ± 2.35 kg m^−2^). All players were training in a soccer club for at least 4 years. Physical sessions of different teams consisted of speed, agility, quickness training, injury prevention or coordination exercises allowing to maintenance physical fitness level. At the time of the study, all players were competing at national and regional level (i.e., Spanish Female Soccer National League, Aragón Female Soccer Territorial League). According to the soccer federation rules the players were grouped on the basis of chronological age into 2 years age categories [U14 (12–13.9 year), U16 (14–15.9  year) and U18 (16–17.9  year)] rather than biological maturation. Female players were divided by age group, in the same way as they are pooled during training and competition.

Participants were healthy and they did not suffer any disease or injury that could affect the results at the moment of the study. Parents/guardian informed consent for all players involved in this investigation were obtained. The study was conducted according to the Declaration of Helsinki (2013), and was approved beforehand by the Ethics Committee of Clinical Research from the Government of Aragón (CP19/039, CEICA, Spain).

### Procedures

Participants were assessed within a single testing session on linear sprinting at the end of the second third of the competitive season (March). Test sessions in different teams were taken by the same group of investigators during the entire study to avoid inter-observer variability, at the same time of the day (6 PM to 8 PM) and under the same environmental conditions (~ 22ºC and ~ 20% of humidity). One week prior to the commencement of the study, players performed two familiarization trials to elude any learning effect. Before performing all the tests, a warm-up, consisting of jogging (3 min), lower-limb dynamic strectches (4 min) and high intensity activities (4 min), was performed. Players wore soccer boots in the field tests on artificial grass.

### Anthropometric data

Anthropometric variables included were height, body mass and sitting height. A portable stadiometer with 2.10 m maximum capacity and a 0.1 cm error margin (SECA 225, SECA, Hamburg, Germany) was used to measure height. Moreover, sitting height was evaluated with a purpose built table (SECA 225, SECA, Hamburg, Germany) [26, 27]. A portable body composition analyser with a 200 kg maximum capacity and a ± 0.1 kg error margin (TANITA BC-418 MA; Tanita Corp., Tokyo, Japan) was used to measure body mass. BMI was calculated by dividing weight (kg) by squared height (m^2^).

### Maturity status

Pubertal timing was estimated according to the biological age of maturity of each individual as described by the non-invasive method of Mirwald et al. [28]. The age of peak linear growth (age at peak height velocity (APHV)) is an indicator of somatic maturity representing the time of maximum growth in stature during adolescence. Biological age of maturity (years) was calculated by subtracting the chronological age at the time of measurement from the chronological peak-velocity age [12, 20]. Thus, a maturity age of -1.0 indicates that the player was measured 1 year before this peak velocity; a maturity of 0 indicates that the participant was measured 1 year after this peak velocity.

### Sprint testing

Running speed was assessed by 40 m sprint time with 10 m, 20 m and 30 m split times. Players commenced each sprint from a standing start with their front foot 0.5 m behind the first timing gate. Time was recorded with photoelectric cells (*Microgate, Bolzano, Italy*). One pair of the electronic timing system sensors mounted on tripods was set at 0.75 above the floor and was positioned 3 m apart facing each other on either side of the starting line [29]. Players started when they wanted, thus eliminating reaction time. Also, they were instructed, and verbally encouraged, to give a maximal effort during each sprint. Two trials were separated by at least 3 min of recovery with the best performance used as the final result [30].

Acceleration (s) was evaluated using the time to cover the first 10 m of the 40 m sprint test. Maximum running speed (s) was assessed using a flying 20 m sprint (i.e. last 20 m of the 40 m sprint effort) [11].

### Statistical analyses

Statistical analyses were performed using SPSS for MAC (Version 20.0; SPSS Inc, Chicago, IL, USA). Descriptive statistics (means ± standard deviations) are reported. For the first part of this study, all analyses of variance (ANOVA) were performed on log-transformed data to reduce bias arising from non-uniformity error. A *post-hoc* Bonferroni analysis was used to determine the origin of any differences. Between-group differences according to age category in sprint performance were assessed using analysis of covariance (one-way ANCOVA). Body mass, BMI and APHV were used as covariates in the present study [16]. Cohen’s *d* effect sizes (ES) were also used to compare magnitude differences in sprint variables and covariates between ages categories. The criteria to interpret the magnitude of the ES were: > 0.2 (small), > 0.6 (moderate), > 1.2 (large), and > 2.0 (very large) [31]. Pearson correlation analyses (*r*) were applied to determine the relationships between age, anthropometrics (height, body mass, BMI), maturation and sprint variables. The significance level was set at *p* < 0.05.

## Results

Descriptive statistics for anthropometric characteristics of the 80 adolescent female soccer players of the U14 to U18 age categories are presented in Table 1. Age and APHV except for BMI, showed significant age differences (U18 > U16 > U14; *p* < 0.05). There were significant differences in height among the U18, U16 and U14 (*p* < 0.05) and body mass between the U14 and U18 age groups (*p* < 0.05).

**Table 1:** Subject characteristics

Groups	Age (year)	Years to /from APHV	Height (cm)	Body mass (kg)	BMI	Training practical (h·w^−1^)
U14 n = 20	13.1 ± 0.5*^#^	0.36 ± 0.58*^#^	154.1 ± 7.6*^#^	48.8 ± 7.8^#^	20.6 ± 2.64	~ 3 h (1 technical-tactical sessions + 1 physical sessions)
U16 n = 37	14.2 ± 1.6^^^	2.01 ± 0.98^^^	157.9 ± 6.8	51.8 ± 7.8	20.8 ± 2.35	~ 4 h (1 technical-tactical sessions + 2 physical sessions)
U18 n = 23	16.7 ± 0.6	2.74 ± 0.48	160.2 ± 6.1	56.1 ± 6.5	21.8 ± 2.07	~ 4 h (1 technical-tactical sessions + 2 physical sessions)

Values are mean ± standard deviation. U14: Under 14 years old. APHV: age at peak height velocity; BMI: Body mass index

*Significant difference (*p* < 0.05) between U14 versus U16 players

^#^Significant difference (*p* < 0.05) between U14 versus U18 players

^^^Significant difference (*p* < 0.05) between U16 versus U18 players

The test of between groups effect, regarding the chronological age, showed significant differentes in 10 m, 20 m, 30 m and 40 m times except for 20 m flying (Table 2). *Posthoc* analysis revealed better performance in all split sprint times of older soccer players (U18 and U16) compared with younger category (F: 3.38 to 6.17; *p* < 0.05; ES: 0.64 to 1.33). On the contrary in all split sprint times, there were no significant changes between U16 and U18 (*p* < 0.05; ES: 0.03 to 0.17). Furthermore, 20 m flying showed no age-related differences (*p* > 0.05); ES: 0.30 to 0.92). An ANCOVA was applied to account for the influence of maturation, body mass and BMI on between group differences in the sprint variables (Table 2). There were significant differences for all variables when APHV was controlled for (F: 3.98 to 18.1; *p* < 0.05). However, body mass and BMI were no significant covariates (*p* > 0.05) for all variables between age groups, except for 20 m flying within U14 and U18 when body mass were statiscally controlled for (F: 5.73; *p* < 0.05).

**Table 2:** Times for 10-m, 20-m, 30-m and 40-m sprint for the U14, U16 y U18 soccer players (mean ± standard deviation), together with effect size differences and results of ANOVA and analysis of covariance (ANCOVA) controlling age peak height velocity, body mass and body mass index

Variables	Age groups			ANOVA		Bonferroni’s post hoc test/ ES (magnitude of difference)	ANCOVA (APHV) F/*p*	ANCOVA (BM) F/*p*	ANCOVA (BMI) F/*p*
	U14	U16	U18	F	*p*				
10 m sprint (s)	2.12 ± 0.10	2.04 ± 0.09	2.02 ± 0.12	4.443	< 0.05*	1–2 (p < 0.05)* (ES: 0.89) 2–3 (p > 0.05) (ES 0.03) 1–3 (p < 0.05)* (ES 0.93)	1–2 F: 9.99 (p < 0.01)** 2–3 F: 4.72 (p < 0.05)* 1–3 F: 12.8 (p < 0.01)**	1–2 F: 0.36 (p > 0.05) 2–3 F: 0.07 (p > 0.05) 1–3 F: 0.52 (p > 0.05)	1–2 F: 0.39 (p > 0.05) 2–3 F: 0.07 (p > 0.05) 1–3 F: 0.54 (p > 0.05)
20 m sprint (s)	3.67 ± 0.14	3.55 ± 0.15	3.52 ± 0.18	3.626	< 0.05*	1–2 (p < 0.05)* (ES 0.69) 2–3 (p > 0.05) (ES 0.09) 1–3 (p < 0.05)* (ES: 0.93)	1–2 F: 10.9 (p < 0.01)** 2–3 F: 6.61 (p < 0.05)* 1–3 F: 3.04 (p > 0.05)	1–2 F: 0.05 (p > 0.05) 2–3 F: 0.03 (p > 0.05) 1–3 F: 0.07 (p > 0.05)	1–2 F: 1.10 (p > 0.05) 2–3 F: 0.51 (p > 0.05) 1–3 F: 1.31 (p > 0.05)
30 m sprint (s)	5.18 ± 0.19	5.02 ± 0.24	4.96 ± 0.26	3.380	< 0.05*	1–2 (p < 0.05)* (ES:0.64) 2–3 (p > 0.05) (ES: 0.11) 1–3 (p < 0.05)* (ES: 0.92)	1–2 F: 10.4 (p < 0.01)** 2–3 F: 6.51 (p < 0.05)* 1–3 F: 11.4 (p < 0.01)**	1–2 F: 0.10 (p > 0.05) 2–3 F: 0.14 (p > 0.05) 1–3 F: 0.03 (p > 0.05)	1–2 F: 1.14 (p > 0.05) 2–3 F: 0.04 (p > 0.05) 1–3 F: 1.39 (p > 0.05)
40 m sprint (s)	6.84 ± 0.27	6.54 ± 0.34	6.41 ± 0.34	6.169	< 0.01**	1–2 (p < 0.05)* (ES: 0.85) 2–3 (p > 0.05) (ES: 0.17) 1–3 (p < 0.01)** (ES: 1.33)	1–2 F: 12.5 (p < 0.01)** 2–3 F: 6.38 (p < 0.05)* 1–3 F: 18.1 (p < 0.01)**	1–2 F: 0.71 (p > 0.05) 2–3 F: 0.04 (p > 0.05) 1–3 F: 0.85 (p > 0.05)	1–2 F: 0.94 (p > 0.05) 2–3 F: 0.04 (p > 0.05) 1–3 F: 1.16 (p > 0.05)
20 m flying (s)	3.11 ± 0.13	2.82 ± 0.64	2.61 ± 0.75	1.695	0.12	1–2 (p > 0.05) (ES: 0.62) 2–3 (p > 0.05) (ES: 0.30) 1–3 (p > 0.05) (ES: 0.92)	1–2 F: 4.79 (p < 0.05)* 2–3 F: 3.98 (p < 0.05)* 1–3 F: 4.34 (p < 0.05)*	1–2 F: 2.33 (p > 0.05) 2–3 F: 0.35 (p > 0.05) 1–3 F: 5.73 (p < 0.05)*	1–2 F: 0.26 (p > 0.05) 2–3 F: 0.02 (p > 0.05) 1–3 F: 0.41 (p > 0.05)

ES: effect size; APHV: age peak height velocity; BM: body mass; BMI: body mass index; 1–2: U14 versus U16, 2–3 U16 versus U18; 1–3 U14 versus U18

*Significant *p* < 0.05

**Significant *p* < 0.01

From the data pooled across all age groups, the relationships between age, anthropometric (height, body mass, BMI), maturation and sprint variables are shown in Table 3. BMI were significantly related to 40 m (*p* < 0.05; *r* = − 0.27). Body mass produced significant correlation (*p* < 0.01; *r* = − 0.38) with 20 m flying. The rest of sprint variables (10 m, 20 m or 30 m) were not significantly related to any of age, anthropometrics ormaturation variables (*p* > 0.05; *r* = − 0.25 to 0.09).

**Table 3:** Pearson’s correlation between age, anthropometric (height, body mass, BMI), maturation and sprint variables of adolescent female soccer players

	Age	Height	Body mass	BMI	APHV
10 m sprint	− 0.128	− 0.085	0.092	− 0.045	− 0.059
20 m sprint	− 0.068	− 0.083	0.015	− 0.236	− 0.142
30 m sprint	− 0.091	− 0.080	− 0.001	− 0.252	− 0.159
40 m sprint	− 0.234	− 0.174	− 0.058	− 0.270*	− 0.243
20 m flying	− 0.272	− 0.066	− 0.375**	− 0.068	− 0.209

APHV: Age at peak height velocity; BMI: Body mass index

*Denotes significant Pearson`s correlation coefficient at the level 0.05 level

**Denotes significant Pearson`s correlation coefficient at the level 0.01 level

## Discussion

The aim of this investigation was to determine differences among speed qualities relevant for soccer performance at different ages, and to examine what possible factors determining sprint performance in adolescent female soccer players. In general, players improved their anthropometric characteristics and their results in sprint test as older they are. Outcomes demonstrated that BMI and body mass were significantly correlated with 40 m sprint and 20 m flying, respectively. Furthermore, in the present study, significant differences were found among groups, specifically in 10 m, 20 m, 30 m and 40 m times. However, when the effect of body mass and BMI was removed, all of the differences disappeared. Conversely, differences between group did not disappear in sprint running performances when adjusted for APHV.

An analysis of the differences between groups revealed improvements in anthropometric characteristics and sprint performance (Table 1 and 2). The current findings demonstrate that these characteristics are developed as age increases. Previous literature has also reported improvements in anthropometric and sprint performance in adolescent female soccer players [25] likely due to biological development and skill acquisition for participation in sport specific soccer training [32]. All split times were faster in older players. The greatest significant changes in speed were observed within U14 and U16/U18. This is probably caused by an increases of height and hence, an increase of stride lenght, just as central nervous system adaptation, which happens around this age [33]. In addition, age related enhancements in sprint performance likely to be associated with the grater glycolitc capacity, type II fibres recruitment capacity and peak lactate concentrations during adolescence [34]. However, insignificant differences were observed between U18 and U16 groups in sprint times. It seems logical to assume that the performance of females players reach a plateau around the age of 13 years (puberty) [35], for this reason it can be a similar performance in sprint times between U16 and U18 groups. Therefore, soccer coaches’ concers about how to train sprint ability, which is related to determinant actions during match (e.g. to score a goal), should be emphasized during adolescence.

Previous studies have evaluated the possible role of growth (i.e. weight, height or body mass) and maturation on different running speed qualities relevant to soccer performance in soccer players [16, 19, 36]. In the present study, all between groups differences in sprint performance disappeared when the body mass and BMI was statiscally controlled. Notwithstanding, the inclusion of the estimation of maturity status (i.e., APHV) as a covariate is significant. These results demonstrated that sprint differences between groups do not exis when the body mass and BMI effect is removed, which can mean that sprint differences are related to anthropometric characteristics. Several studies have observed that male soccer players are being chosen due to their maturity level instead of their physical performance or anthropometric characteristics [16, 37], but this is the first study to analyse it in adolescent female soccer players. Malina et al. [38] showed that anthropometric factors (weight and height) compared to sexual maturity, are significant contributors to sprint in male soccer players between 13 and 15 years. Mendez-Villanueva et al. [16] showed that maturation explained a greater proportion of age-related differences in speed capacities than body dimensions in young male soccer players. This discrepancy in observations for male players is likely related in part with maturing measure used. The estimated maturity was assessed by stage of pubic hair and APHV, respectively. The use of the Mirwald equation to assess maturity status has been previously questioned due to its potential associated error of ± 6 months [39]. Therefore, caution must be taken into account when comparing both methods. So, it is not currently clear what covariates explain age-realted differences in speed and what method is the gold standard. This suggests that the age-related differences in sprint running performance in the present study were more related to differences in anthropometric rather than differences in maturation factors per se.

Different studies have analyzed anthropometric profile of soccer players [40, 41], they found that height and body mass were relevant factors in soccer performance. specifically, height and body mass were associated with sprint performance in youth soccer players [16]. The results indicated that BMI and body mass were significantly correlated with 40 m sprint time (*p* < 0.05) and 20 m flying (*p* < 0.01). These findings were consistent with previous investigations in young soccer players [42–44]. Mathisen et al. and Wong et al. observed significant correlations between body mass with 10 m and 30 m sprint performance among adolescent male soccer players [42, 43]. Moreno et al. found that body fat, which should be connected to BMI, correlated significantly with sprint performance [44]. Conversely, previous studies observed insignificant relationship between anthropometric and sprint performance [7, 45, 46]. Therefore, our results can be explained by antropometric characteristics, which might suggest that sprint performance is related to variation in body mass and BMI. However, there are limited data in adolescent female soccer players and there is a lack of consisten results for sprint performance.

A possible limitation of the present study was that the results have a cross-sectional character and concern a number of age groups with a relatively small size. Second, it is possible that resistance training can affect speed development, however, resistance training has not been controlled during the training courses. Furthermore, the results concern adolescent female soccer players. Consequently, they cannot be directly extrapolated to male players and athletes at other skill levels. In addition, female players were grouped by chronological age and comparisons among groups did not take into account the biological maturity of female players. Finally, the playing position of sample were not assessed within the study. Future studies should analyse possible differences in sprint and other performance capacities in regard to maturational status of players, tactical position and playing positions in female soccer players.

## Conclusions

To conclude, this study emphasized that players may improve their anthropometric characteristics and their results in sprint tests as older they are. Also, significant differences between younger and older age categories were observed because younger players were far to their age of peak competitive performance. We suggest that the age effects on running speed qualities during growth in the current cohort of female adolescent soccer players were likely related to anthropometric factors rather than biological maturation per se. This idea is also reinforced by the fact that when body mass and BMI are considered as covariates, all sprint variables were very similar among the studied groups.

## Data Availability

The datasets generated during and analyzed during the current study are available from the corresponding author on reasonable request.

## Abbreviations

APHV: Age at peak height velocity
BMI: Body mass index
